# A Governance-Aware Private Cloud Architecture for Scalable Multi-Provider Vehicle-Based Multimodal Sensing

**DOI:** 10.3390/s26061939

**Published:** 2026-03-19

**Authors:** Zdravko Kunić, Vedran Dakić, Zlatan Morić

**Affiliations:** 1 Academic Unit for Information Systems and Business Analytics, Algebra Bernays University, Gradišćanska ulica 24, 10000 Zagreb, Croatia; 2 Academic Unit for System Engineering and Cybersecurity, Algebra Bernays University, 10000 Zagreb, Croatia; zlatan.moric@algebra.hr

**Keywords:** vehicle-mounted mobile sensing, multimodal data collection, private cloud architecture, Internet of Things (IoT), smart city monitoring

## Abstract

Vehicle-mounted sensing enables high-resolution urban monitoring but remains constrained by heterogeneous multimodal integration, intermittent connectivity, privacy-sensitive visual data, and the absence of enforceable multi-provider governance. This paper introduces a governance-aware private cloud architecture that treats provider isolation, role-based access control, and privacy-by-design as core architectural properties rather than application-layer add-ons. The layered, containerised microservice design supports asynchronous store-and-forward ingestion, modality-specific processing pipelines, and GPU-accelerated object detection for structured metadata extraction. A key innovation is ingestion-time visual abstraction, which structurally separates raw imagery from derived observations and enforces lifecycle-based retention policies, embedding data minimisation directly into the data flow. The fully open-source implementation is validated through a two-month multi-provider pilot with continuous multimodal collection. Results demonstrate stable ingestion without data loss, real-time visual inference (~200 ms per frame), strict provider-level isolation under concurrent access, and up to 95% storage reduction via metadata abstraction. The findings establish a replicable architectural paradigm for scalable, privacy-aware, multi-actor mobile sensing infrastructures suitable for metropolitan-scale smart city deployment.

## 1. Introduction

Advances in low-cost environmental sensors, embedded computing, and mobile connectivity have transformed vehicles into mobile sensing platforms capable of high-resolution urban monitoring. By leveraging existing fleets—such as delivery vehicles, public transport, and municipal service units—vehicle-mounted sensing systems provide spatial coverage and temporal density that static infrastructures cannot economically achieve. These systems support applications including environmental surveillance, traffic analysis, infrastructure assessment, and smart city governance.

However, large-scale operational deployment remains limited not by sensing capability but by architectural constraints. Vehicle platforms generate heterogeneous multimodal streams—meteorological, air quality, acoustic, geospatial, and visual—under intermittent connectivity and asynchronous transmission conditions. Simultaneously, real-world deployments involve multiple independent stakeholders with distinct data ownership, access rights, and regulatory obligations. Existing solutions address sensing performance, cloud scalability, or privacy techniques in isolation, but do not integrate asynchronous multimodal ingestion, enforceable multi-provider governance, and structural privacy-by-design within a unified architecture. As a result, governance is often contractual rather than technical, and privacy protection remains post hoc rather than embedded in the data lifecycle.

This architectural disconnect inhibits scalable, multi-actor mobile sensing in practice. Notwithstanding considerable advancements in mobile sensing and IoT cloud platforms, the integration of governance, privacy preservation, and heterogeneous multimodal sensing remains disjointed within current system architectures. Most contemporary systems prioritise either sensing performance, infrastructure scalability, or privacy approaches, seldom considering these elements as coequal design constraints within a cohesive system paradigm. Multi-provider installations pose intricate problems in data ownership, access control, and regulatory compliance, which are typically resolved through organisational agreements rather than enforceable system-level mechanisms. As a result, there is a lack of architectural frameworks that concurrently support asynchronous multimodal sensing, separation of structural providers, and privacy-conscious data processing in operational vehicle-based sensing infrastructures. The fundamental motivation of this study is to address the integration gap.

To address this gap, we propose a governance-aware private cloud architecture that embeds provider isolation, role-based access control (RBAC), and data minimisation as enforceable architectural constraints rather than application-layer policies. Governance spans ingestion, storage, and API layers, ensuring provider-scoped segmentation and lifecycle-wide policy enforcement. The layered, containerised microservice design supports asynchronous store-and-forward ingestion and modality-specific processing pipelines, while GPU-accelerated object detection enables the extraction of structured metadata from visual streams.

A key architectural innovation is visual abstraction during ingestion. Raw image frames are processed immediately upon arrival, converted into structured detection metadata, and subjected to retention policies that minimise long-term storage of identifiable content. By separating raw imagery from derived observations at the architectural level, the system operationalises privacy-by-design directly within the data flow, reducing regulatory exposure and storage overhead without compromising analytical utility. The study addresses three research questions:

- RQ1: How can a private cloud architecture reliably support asynchronous, heterogeneous, multimodal vehicle-mounted sensing across multiple independent providers?
- RQ2: How can provider-level governance and isolation be enforced structurally within core architectural layers rather than delegated to application logic?
- RQ3: How can privacy-sensitive visual streams be managed through ingestion-time abstraction instead of post hoc anonymisation?

This work makes three primary architectural contributions:

1. Governance-embedded private cloud architecture: We introduce a multi-tenant private cloud design in which provider isolation, role-based access control (RBAC), and policy enforcement are embedded as cross-layer architectural constraints rather than application-layer controls. Governance mechanisms span the ingestion, storage, and API layers, ensuring the structural separation of data ownership and the enforcement of provider-scoped access throughout the system lifecycle.
2. Ingestion-time visual abstraction and data minimisation framework: We propose a processing pipeline that operationalises privacy-by-design at the data-flow level by separating raw visual imagery from structured detection metadata during ingestion. Unlike post hoc anonymisation approaches, this architecture enforces data minimisation before long-term storage, substantially reducing the retention of identifiable content while preserving analytical utility.
3. Operational validation of governed, asynchronous multimodal sensing: The proposed architecture is implemented using a fully containerised, open-source technology stack and validated through a sustained multi-provider vehicle-based deployment. Empirical evaluation demonstrates reliable asynchronous ingestion under intermittent connectivity, real-time GPU-accelerated visual inference, enforceable provider-level isolation under concurrent access, and significant storage reduction through ingestion-time abstraction.

Collectively, these contributions transform governance and privacy from policy-level considerations into enforceable architectural primitives embedded within the data flow of multi-provider vehicle-mounted sensing systems.

The remainder of this paper is structured as follows: Section 2 reviews related work. Section 3 presents the system architecture and design principles. Section 4 details acquisition, processing, and governance mechanisms. Section 5 describes implementation and performance evaluation. Section 6 outlines application scenarios. Section 7 discusses limitations and future work. Section 8 concludes the paper.

## 2. Related Work

This section examines prior studies on vehicle-mounted mobile sensing systems and IoT-based urban monitoring frameworks. The research landscape is delineated into three analytical perspectives: (i) vehicle-based sensing platforms and data acquisition methodologies, (ii) multimodal sensing and data fusion techniques, and (iii) IoT cloud architectures and governance frameworks facilitating extensive sensing infrastructures. This classification underscores the complementary yet predominantly isolated research streams that currently define the subject and establishes the foundation for recognising the architectural integration gap that this study addresses.

While prior research has established the feasibility of mobile sensing and IoT cloud infrastructures, most existing systems are either application-specific prototypes or assume centralised single-provider governance. Few works address architectural multi-tenancy, provider isolation, and lifecycle-integrated privacy enforcement as core system design principles. This review, therefore, evaluates prior systems through the lenses of scalability, governance integration, and privacy-aware multimodal processing.

### 2.1. Mobile Sensing on Vehicle Platforms

Vehicle-mounted mobile sensing, commonly known as drive-by or opportunistic sensing, has become a cost-effective means of collecting high-resolution urban data by leveraging existing vehicle fleets. Preliminary studies have demonstrated the feasibility of using taxis, buses, and service vehicles as mobile sensor platforms for monitoring air quality, noise, and road conditions [1,2,3]. In contrast to static sensor networks, mobile platforms provide greater spatial coverage and greater adaptability to dynamic urban environments [4].

Numerous studies have examined the sensing efficacy and optimisation of vehicle-based sensing fleets, focusing on route coverage, sampling frequency, and fleet composition [5,6]. Zheng et al. conducted an extensive survey on urban drive-by sensing, emphasising the correlation between sensing performance and mobility patterns [7]. Recent research has broadened its scope from environmental sensing to encompass traffic characterisation, infrastructure evaluation, and visual analytics utilising onboard cameras [8,9,10]. Recent studies have examined optimization strategies for vehicle fleet deployment and sensing coverage in extensive urban monitoring contexts, emphasizing the significance of mobility patterns and vehicle scheduling in enhancing spatial-temporal sensing efficacy [11,12]. Also, the latest implementations increasingly regard vehicles as distributed sensor nodes that can monitor urban infrastructure and environmental conditions in real time [13]. Nevertheless, most current research emphasises discrete sensing tasks and lacks comprehensive system architectures that support sustained operation, multimodal data integration, and governance among diverse stakeholders.

### 2.2. Multimodal Data Collection

Multimodal data acquisition integrates diverse sensor streams—such as meteorological, air quality, acoustic, and visual data—to enhance situational awareness and analytical depth. Recent efforts have concentrated on creating extensive multimodal vehicle-mounted datasets that include LiDAR, panoramic photography, GNSS/INS navigation data, and supplementary sensors to facilitate improved perception and urban mapping studies [14]. Previous studies have demonstrated that integrating various sensing modalities can markedly improve robustness and interpretability in urban monitoring applications [15,16]. Mobile platforms equipped with cameras facilitate object detection, traffic analysis, and the contextual enhancement of sensor measurements [17].

Notwithstanding these advancements, multimodal systems present challenges related to synchronisation, asynchronous data influx, storage heterogeneity, and privacy protection [18]. Numerous frameworks propose sensor fusion pipelines or edge-assisted processing to mitigate bandwidth and latency constraints [19,20]. Nonetheless, numerous implementations are application-specific, tightly coupled to particular sensing configurations, and offer minimal support for extensibility or independent data providers. The systematic management of raw and derived data, particularly for privacy-sensitive visual streams, remains an unresolved research challenge.

### 2.3. Cloud Architectures for Mobile Sensing in the Internet of Things

IoT paradigms provide the architectural foundation for scalable mobile sensing systems by enabling distributed data ingestion, device abstraction, and service interoperability. Cloud-based IoT frameworks have been extensively studied for smart city applications, enabling large-scale sensor deployments and real-time data access [21,22]. Recent studies underscore the necessity for secure-by-design IoT designs that incorporate privacy, security, and governance elements into the system architecture from the initial design phases [23]. Studies have highlighted the significance of microservices, message brokers, and containerisation in attaining scalability and fault tolerance [24].

Numerous studies advocate for stratified IoT architectures that delineate sensing, data transmission, storage, and application services, thereby enhancing modularity and long-term sustainability [25,26]. Recent research underscores the significance of governance, access control, and data ownership in multi-actor IoT ecosystems, especially when data is sourced from independently managed devices and fleets [27,28]. Despite advances in microservices and containerisation, most IoT cloud architectures are designed for static sensor deployments or single-tenant environments.

### 2.4. Data Governance and Privacy in Mobile IoT Sensing Systems

The widespread deployment of mobile IoT sensing systems poses significant challenges for data governance, privacy protection, and regulatory compliance. In contrast to static sensor networks, vehicle-mounted platforms consistently collect data across diverse spatial contexts, often obtaining information that may be sensitive, indirectly identifiable, or subject to regulatory constraints [29]. These challenges are exacerbated when sensing involves visual data, locational traces, or information from multiple independent contributors.

Previous research has highlighted the importance of privacy-conscious system design in IoT settings, particularly regarding data minimisation, anonymisation, and access control [30]. Recent studies investigate privacy-preserving protocols and cross-layer security mechanisms that integrate cryptographic methods, federated learning, and secure aggregation to safeguard sensitive data within IoT and smart city frameworks [31]. In mobile sensing contexts, unprocessed data streams—such as high-definition video or precise geolocation data—can compromise the privacy of individuals or assets, even when collected for legitimate surveillance purposes. Consequently, numerous studies endorse architectural methodologies that distinguish between raw data collection and processed or aggregated representations to mitigate privacy risks while maintaining analytical integrity [32].

Data governance is notably complex in multi-provider IoT ecosystems, where data ownership, usage rights, and responsibilities are distributed among stakeholders, including fleet operators, platform owners, service providers, and end users [33]. Recent research on smart-city governance frameworks underscores the need for explicitly delineated responsibilities, access protocols, and accountability mechanisms to ensure data ownership and reliable multi-stakeholder collaboration [34]. Current IoT frameworks often assume centralised ownership or single-operator control, limiting their applicability in collaborative or federated sensing contexts. The absence of clear governance mechanisms can undermine trust, scalability, and regulatory compliance, including under the General Data Protection Regulation (GDPR).

Role-based access control (RBAC) and policy-driven data management have been proposed as essential mechanisms to mitigate these challenges in IoT systems [35]. Nonetheless, numerous implementations remain theoretical or are utilised solely at the application layer, lacking integration into the fundamental data ingestion and processing pipelines. This division diminishes transparency and complicates the enforcement of data-use policies throughout the system’s lifecycle.

Existing privacy approaches predominantly rely on anonymisation, aggregation, or cryptographic safeguards applied after data collection. Architectural enforcement of data minimisation at ingestion time remains comparatively underdeveloped, particularly for continuous visual sensing streams.

### 2.5. Large-Scale Urban Sensing and Community-Based Mobile Sensing Platforms

Several large-scale urban sensing initiatives have explored distributed data-collection infrastructures with varying emphases on openness, governance, and deployment modalities. The Array of Things (AoT) project [36] is among the most mature open-source urban sensing platforms deployed in a metropolitan environment. AoT integrates modular sensor nodes installed on fixed urban infrastructure (e.g., light poles) to collect environmental and mobility-related data. The project emphasises open data principles, privacy-aware processing at the node level, and public transparency regarding data governance. However, AoT relies on static infrastructure rather than mobile platforms, which limits its spatial adaptability compared to vehicle-mounted sensing. Furthermore, governance in AoT is primarily oriented toward municipal-level oversight and public data dissemination rather than toward structural isolation of multiple providers within a shared architectural framework.

The SmartSantander testbed [37] was among the earliest large-scale city IoT experimentation platforms, deploying thousands of heterogeneous sensors across urban environments. The project focused on validating IoT scalability and interoperability and on deploying experimental services in a real-world setting. While SmartSantander demonstrated the feasibility of large-scale sensor federation and heterogeneous device management, it largely assumed centralised governance and fixed sensor deployments. Multi-provider architectural isolation and lifecycle-integrated privacy enforcement were not primary design constraints.

The OpenSense project [38] introduced a community-based mobile environmental sensing approach in which sensors mounted on public vehicles collected air quality data across urban areas. OpenSense demonstrated the advantages of leveraging mobility to enhance spatial coverage and addressed sensor calibration challenges in mobile settings. However, the system architecture primarily focused on data acquisition and environmental modelling rather than embedding multi-provider governance or architectural privacy-by-design mechanisms across the data lifecycle.

Similarly, the MetroSense initiative [39] explored people-centric mobile sensing using personal mobile devices as distributed data collectors. MetroSense investigated large-scale participatory sensing architectures and addressed scalability, data heterogeneity, and participant incentives. Nevertheless, governance mechanisms were conceptual and application-layer-oriented, lacking structural provider-level isolation or ingestion-time privacy abstractions for multimodal visual data streams.

### 2.6. Deficiencies in Existing Research

Despite substantial progress in vehicle-mounted sensing, multimodal data acquisition, and IoT cloud architectures, existing research remains structurally fragmented along three principal dimensions.

First, vehicle-based sensing systems predominantly emphasise sensing performance, spatial coverage, and analytical optimisation. While these works demonstrate the feasibility and utility of mobile data collection, governance is rarely treated as an architectural constraint. Multi-provider isolation, enforceable data ownership boundaries, and lifecycle-wide access control are typically treated as organisational or contractual matters rather than as embedded structural properties of the system itself.

Second, multimodal sensing frameworks enhance analytical robustness through sensor fusion and contextual enrichment; however, most implementations lack extensible architectures that support heterogeneous providers and asynchronous ingestion. Although scalability is often addressed at the infrastructure level, fine-grained provider-level governance and the architectural separation of raw and derived data remain underexplored in vehicle-mounted multimodal contexts.

Third, privacy-preserving approaches in mobile IoT sensing are predominantly post hoc. Techniques such as anonymisation, aggregation, or cryptographic safeguards are typically applied after data collection and storage. Architectural enforcement of data minimisation at the ingestion stage—particularly for continuous visual streams—remains comparatively underdeveloped. As a result, raw identifiable data are frequently retained longer than operationally necessary, increasing both storage overhead and regulatory exposure.

Table 1 presents exemplary systems examined in the literature, highlighting many architectural and functional features pertinent to extensive vehicle-based sensor infrastructures. The chosen criteria represent the essential system-level capabilities investigated in this study, encompassing support for vehicle-mounted sensors, multimodal data integration, asynchronous data ingestion, multi-provider governance, role-based access control, and privacy-conscious processing of visual data. The comparison does not seek to deliver a comprehensive assessment of all system characteristics, but rather emphasises whether these capabilities are expressly incorporated into the architectural design of the cited systems. A checkmark (Y) signifies explicit support, a dash (–) suggests that the feature is not addressed, and a circle (o) represents partial or indirect support.

The comparison reveals that current systems generally focus on only specific portions of these architectural dimensions. Numerous vehicle-based sensing technologies focus primarily on data collection and environmental monitoring, whereas IoT cloud architectures prioritise scalability and service adaptability. However, integrated support for asynchronous multimodal ingestion, enforceable multi-provider governance, and privacy-aware visual processing remains largely absent. The suggested architecture distinguishes itself from previous systems by treating these issues as coequal architectural design constraints rather than discrete system features.

The literature research consequently exposes a distinct gap in architectural integration. Current research predominantly concentrates on certain aspects of mobile sensing systems: sensing efficacy and fleet optimisation, multimodal data collection and analysis, or cloud-based IoT frameworks. Nevertheless, these study domains seldom coalesce into a cohesive architectural framework that can simultaneously facilitate asynchronous multimodal intake, enforce multi-provider governance, and ensure privacy-conscious data processing.

This paper proposes an architecture that bridges this gap by including three dimensions into a governance-aware private cloud platform tailored for vehicle-based multimodal sensing deployments with numerous independent data providers.

## 3. System Architecture and Design Principles

This section presents the overall architecture of the proposed private cloud platform for asynchronous multimodal data collection from vehicle-mounted sensors. The design is guided by five foundational principles: modularity, scalability, asynchronous operation, multi-provider governance, and privacy by design. These principles collectively ensure that the system can accommodate heterogeneous data sources, support multiple independent stakeholders, and maintain regulatory compliance throughout the data lifecycle.

### 3.1. Design Principles

The architecture is grounded in a modular, layered design that separates concerns across distinct functional domains. Each layer encapsulates a specific set of responsibilities: data acquisition, ingestion, processing, storage, governance, and application services. This separation enables independent development, testing, and scaling of individual components without affecting the broader system. Furthermore, adherence to open standards and open-source technologies throughout the stack ensures long-term maintainability and reduces vendor dependence, both of which are essential for research-oriented and public-sector deployments.

Unlike conventional IoT cloud architectures that assume single-tenant deployments, the proposed design treats governance and provider isolation as primary architectural constraints rather than post-deployment controls. This shift alters the system’s trust boundaries and influences data routing, storage segmentation, and API design.

Scalability is addressed through containerised microservice deployment, in which each functional component operates as an isolated service that can be horizontally scaled to meet workload demands. This approach avoids monolithic bottlenecks and enables elastic resource allocation, which is particularly important given the variable, bursty nature of data streams generated by mobile sensing platforms operating across heterogeneous urban environments.

Asynchronous operation constitutes a central design requirement. Vehicle-mounted sensors operate independently and intermittently, transmitting data at irregular intervals depending on connectivity, route coverage, and operational schedules. The architecture, therefore, decouples data production from consumption through message brokering and queue-based ingestion, ensuring that temporary disconnections, transmission delays, or variable upload rates do not compromise data integrity or system availability. This asynchronous paradigm also allows multiple vehicles and providers to submit data concurrently without the overhead of coordination.

Multi-provider governance is embedded at the architectural level rather than treated as an application-layer concern. The platform supports multiple independent data providers, each operating autonomous-vehicle fleets and sensor configurations within a unified administrative framework. Provider-level isolation ensures that data ownership, access policies, and processing rules are enforced consistently and transparently across all system interactions. Administrative boundaries between providers are maintained at every layer, from data ingestion through storage and retrieval, preventing unauthorised cross-provider data access.

Privacy by design guides the treatment of sensitive data, particularly visual streams captured by onboard cameras. Rather than storing raw imagery indefinitely, the architecture incorporates dedicated processing stages that extract structured metadata through object recognition, thereby reducing the volume of privacy-sensitive material while preserving analytical value. This principle aligns with the data minimisation requirements of regulatory frameworks such as the General Data Protection Regulation and ensures that privacy considerations are addressed systematically rather than retrospectively.

### 3.2. Layered Architecture Overview

The proposed system comprises six functional layers, each addressing a distinct aspect of the data lifecycle. These layers are organised hierarchically, progressing from physical data acquisition at the periphery to high-level application services at the platform’s core.

**Figure 1:**
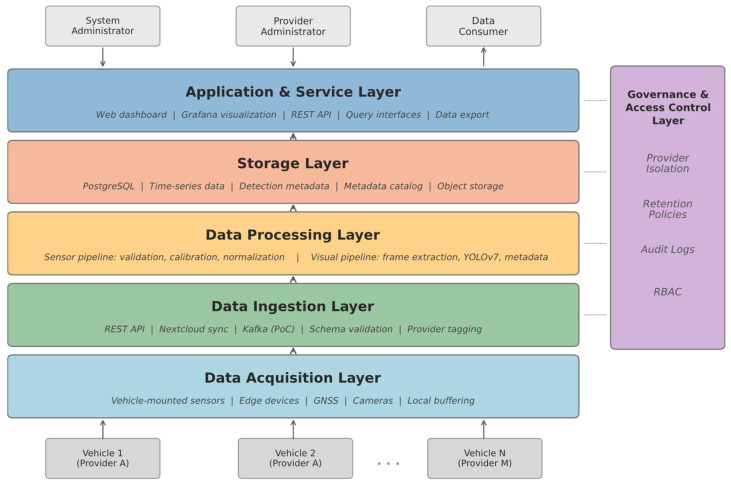
Layered architecture of the proposed private cloud platform for vehicle-mounted multimodal sensing. The six functional layers are organised hierarchically from physical data acquisition at the periphery to application services at the core. Arrows indicate the primary direction of data flow; the governance layer spans all other layers to enforce access control and policy compliance throughout the data lifecycle.

Figure 1 illustrates the overall layered architecture and the data flow between layers.

Trust boundaries exist between (i) vehicle edge devices and cloud ingestion services, (ii) internal microservices, and (iii) external consumers interacting through APIs. All cross-boundary communication is authenticated and encrypted, and no internal service is granted implicit cross-provider visibility.

The data acquisition layer comprises the vehicle-mounted sensor hardware and onboard computing units that capture multimodal data streams. Each vehicle is equipped with meteorological sensors, air quality monitors, acoustic sensors, and one or more cameras. An onboard edge device coordinates local data buffering, preliminary validation, and transmission scheduling. This layer operates autonomously from the central platform, enabling vehicles to collect and temporarily store data independently of network availability, which is essential for uninterrupted operation during extended sensing campaigns.

The data ingestion layer receives incoming data streams from multiple vehicles and providers via a message broker. Asynchronous message queues decouple data producers from downstream processing components, absorbing variations in transmission rates and accommodating concurrent uploads from numerous vehicles. Each incoming data package is tagged with provider identification, device metadata, geospatial coordinates, and timestamps to ensure full traceability throughout subsequent processing stages. The ingestion layer also performs initial schema validation and rejects malformed submissions before they enter the processing pipeline.

The data processing layer applies modality-specific transformations to ingested data. Meteorological, air quality, and acoustic measurements undergo validation, calibration correction, and normalisation to ensure consistency across heterogeneous sensor types and providers. Visual data are processed through object detection pipelines that identify and classify relevant entities—such as vehicles, pedestrians, traffic signs, and infrastructure elements—and convert these observations into structured metadata records. This processing step is essential for reducing long-term storage requirements and mitigating privacy risks associated with retaining raw visual data. Processing tasks are distributed across containerised workers that scale independently based on the volume and modality of incoming data.

The storage layer provides persistent, organised retention of both raw and processed data. Time-series databases support continuous sensor measurements with efficient temporal indexing. At the same time, object storage repositories manage larger binary assets, such as image frames, when retention is mandated by policy or analytical requirements. A metadata catalogue indexes all stored records with provenance information, enabling efficient retrieval based on temporal, spatial, or thematic criteria. The storage design ensures that data from different providers remains logically separated while being queryable through a unified interface, thereby supporting both provider isolation and cross-provider analytics where authorised.

The governance and access control layer implements the role-based administrative model that regulates all interactions with the platform. This layer defines and enforces roles such as system administrator, provider administrator, data manager, and data consumer, each with distinct permissions governing data submission, processing configuration, access, and export. Access control policies are evaluated at each data request, ensuring that authorisation decisions reflect current role assignments and provider-level rules. Technically, policy enforcement is implemented in the REST API middleware layer, where authenticated user tokens (e.g., JWTs) encode provider and role identifiers. Each request is validated against a centralised policy engine before query execution. Provider-level isolation is enforced using a combination of provider-scoped database schemas and API-layer filtering based on authenticated token claims (provider_id, role_id). Queries are dynamically constrained to the requesting provider’s namespace, and row-level security policies prevent cross-provider access even in the event of application-layer misconfiguration. The governance layer also maintains comprehensive audit logs of all data access and administrative actions, supporting accountability, dispute resolution, and regulatory compliance. This centralised policy enforcement ensures that governance is applied uniformly across all system components rather than being delegated to individual services.

The application and service layer exposes platform functionality to external users and systems through standardised application programming interfaces. Data consumers interact with the platform through query interfaces that support spatial, temporal, and attribute-based filtering of sensor measurements and derived metadata. Visualisation services, analytical dashboards, and data export tools are provided as modular application components that consume data exclusively through the governed API layer, ensuring that all external access is subject to the permissions and policies defined in the governance model. This design permits the development of diverse client applications without requiring modifications to the underlying platform services.

### 3.3. Integration and Deployment Considerations

The architecture is designed for deployment on private cloud infrastructure using container orchestration platforms. All services are packaged as container images and managed through declarative configuration, enabling reproducible deployments across different hardware environments. Service discovery, load balancing, and health monitoring are handled by the orchestration layer, reducing operational complexity and supporting automated recovery from individual component failures.

Inter-service communication follows an event-driven pattern, with message brokers mediating data flow between ingestion, processing, and storage components. This design minimises tight coupling between services and permits individual components to be updated, scaled, or replaced without system-wide disruption. Configuration management and secret handling are centralised to ensure consistent security policies across all deployed services.

The modular architecture enables incremental deployment, allowing operators to activate additional processing pipelines, storage backends, or application services as operational requirements evolve. This extensibility is particularly valuable in research and pilot deployment contexts, where sensing configurations, analytical objectives, and the number of participating providers may change over the course of a deployment campaign. The layered design further ensures that platform-level architectural decisions do not constrain the diversity of sensing hardware or analytical methods that may be integrated in the future.

Architecturally, the proposed system differs from conventional IoT cloud stacks in three respects: (i) governance logic spans all architectural layers, (ii) privacy-sensitive visual abstraction is enforced at ingestion time rather than post hoc, and (iii) provider isolation is embedded structurally rather than contractually. These characteristics distinguish the platform from typical microservice IoT deployments.

### 3.4. Architectural Design Rationale

This study’s architectural design illustrates critical trade-offs inherent in extensive mobile sensing infrastructures that incorporate heterogeneous devices and multiple independent data suppliers. The main aim of the architecture is not only to facilitate multimodal data input but also to impose governance and privacy limitations as inherent aspects of the system.

A crucial design decision pertains to the distinction between unprocessed visual data and generated information. Numerous sensing platforms preserve unprocessed imagery and implement anonymisation or filtering during subsequent processing phases. Conversely, the suggested architecture executes object detection promptly following ingestion and transforms visual observations into structured information records. This design diminishes the prolonged retention of potentially identifiable visual content and directly implements the notion of data minimisation.

A secondary design aspect pertains to multi-provider governance. Traditional IoT cloud architectures generally assume a single administrative domain and treat access control as an application-layer attribute. Conversely, the current solution implements provider-level isolation across both the API and database layers, ensuring that governance requirements are enforceable despite potential application-level misconfigurations.

A third design trade-off pertains to asynchronous data ingestion. Vehicle-mounted sensing systems operate with intermittent connectivity, rendering synchronous ingestion architectures impractical in practice. The architecture utilises message-based and store-and-forward ingestion mechanisms that separate data production from subsequent processing services. This architecture enhances robustness to connectivity outages while enabling simultaneous data ingestion from multiple independent vehicles.

The containerised microservice architecture was chosen to facilitate modular scalability and the independent development of system components. Instead of utilising a monolithic sensing platform, the design permits independent scaling or replacement of specific processing services—such as visual inference pipelines or sensor calibration modules—without affecting other components. This versatility is especially crucial in research-focused implementations where sensing modalities, analytical models, and governance requirements may change over time.

## 4. Data Acquisition Mechanisms, Processing Pipelines, and Governance Framework

This section describes the operational mechanisms for acquiring multimodal data from vehicle-mounted sensors, the processing pipelines that transform raw measurements into analytically useful representations, and the governance framework that governs data ownership, access, and privacy throughout the system. Whereas Section 3 presented the architectural principles and layered structure, this section addresses the functional details of data flow, transformation logic, and administrative policy enforcement. The system can be understood as a multi-stage data transformation pipeline that progressively converts raw multimodal observations into structured analytical records. Each phase of the pipeline executes a distinct function: acquisition gathers raw measurements from diverse sensing devices; ingestion authenticates and contextualises incoming data streams; processing converts raw inputs into standardised representations and derived metadata; and governance mechanisms implement ownership, access control, and lifecycle policies for all stored data. This pipeline-oriented perspective elucidates how the architectural layers outlined in Section 3 are translated into practical data flows within the platform.

### 4.1. Data Acquisition Mechanisms

Data acquisition in the proposed platform is organised around sensing sessions. A sensing session is initiated when a vehicle begins a data-collection route and is terminated upon completion or upon loss of connectivity after a configurable timeout. During each session, the onboard edge device continuously polls attached sensors and cameras at modality-specific sampling intervals, producing timestamped measurement records that are buffered locally before transmission.

Meteorological sensors capture ambient temperature, relative humidity, atmospheric pressure, and wind speed at intervals typically ranging from one to ten seconds, depending on deployment requirements. Air quality monitors record concentrations of particulate matter, nitrogen dioxide, ozone, and carbon monoxide, with sampling rates governed by sensor response characteristics and calibration protocols. Acoustic sensors capture sound pressure levels and spectral features relevant to noise pollution assessment. Each sensor stream is packaged with device calibration metadata and geospatial coordinates obtained from an onboard GNSS receiver, ensuring that every measurement can be accurately located and contextualised during subsequent analysis.

A calibration and verification mechanism was implemented to guarantee the reliability of measurements in the environmental sensing modules. The initial calibration involved comparing sensor readings with reference measurements from adjacent fixed environmental monitoring stations. In this method, sensor outputs were recorded under controlled conditions, and linear correction functions were established for temperature and humidity readings. Subsequent periodic verification was performed during field deployment by comparing aggregated readings with publicly accessible environmental data from municipal monitoring stations. This comparison facilitated the detection of systematic discrepancies and possible sensor drift. Minor discrepancies were rectified utilising calibration factors implemented during the data input phase of the processing pipeline. The mobile sensing context introduced unpredictability due to spatial movement and microenvironmental effects; nonetheless, the calibration approach ensured that the collected measurements remained sufficiently accurate for urban environmental monitoring applications.

Visual data acquisition follows a distinct protocol. Onboard cameras capture image frames at configurable intervals or in response to event-based triggers, such as the detection of predefined geographic zones or significant changes in scene content. Given the substantially higher bandwidth requirements of visual data, the edge device applies preliminary compression and, where computational resources permit, runs lightweight object detection models to generate initial metadata annotations before transmission. This edge-level preprocessing reduces the volume of data transmitted to the central platform and accelerates downstream processing.

Data transmission from vehicles to the central ingestion layer employs a store-and-forward strategy. When network connectivity is available, the edge device transmits buffered data packages in chronological order through authenticated, encrypted channels. In the event of connectivity interruption, packages are retained in local storage and transmitted upon reconnection, preserving temporal completeness without requiring real-time communication. Each data package includes a unique session identifier, provider credentials, device serial numbers, and integrity checksums, enabling the ingestion layer to verify authenticity and detect transmission errors or duplicate submissions.

### 4.2. Processing Pipelines

Upon ingestion, data is routed to modality-specific processing pipelines that perform validation, transformation, and enrichment. The pipeline architecture is designed to accommodate heterogeneous sensor types and to ensure that outputs conform to standardised schemas regardless of the originating hardware or provider.

For meteorological and air quality measurements, the processing pipeline performs range validation to identify and flag anomalous readings that fall outside physically plausible bounds. Calibration correction functions are subsequently applied to compensate for known sensor drift, cross-sensitivity effects, and environmental biases. Temporal alignment routines reconcile minor discrepancies in sampling timestamps across co-located sensors, producing synchronised multimodal records. The processed measurements are stored in time-series databases, with standardised units and quality indicators that reflect the confidence level of each value.

Environmental noise levels are acquired at discrete geographic points along the vehicle route. The onboard sensor records instantaneous sound pressure levels, which are stored alongside the corresponding geospatial coordinates and timestamps. These point-based noise measurements enable spatial mapping of urban sound environments and longitudinal comparison of noise exposure across locations and time periods, without requiring the capture or retention of raw audio waveforms.

The visual data processing pipeline constitutes the most computationally intensive component of the system. Ingested image frames are submitted to object detection models that identify and localise entities of interest within each scene. Detected objects are classified into predefined categories, including vehicles, pedestrians, cyclists, traffic infrastructure, and urban furniture. For each detection, the pipeline records the object class, bounding box coordinates, confidence score, and contextual attributes such as estimated distance and relative position. These structured detection records are stored as metadata entries linked to the originating image frame, the corresponding geospatial coordinates, and the sensing session.

A critical function of the visual processing pipeline is separating structured metadata from raw imagery. Once object detection is complete and metadata has been generated, the platform applies retention policies to determine whether the original image frame is preserved, archived, or deleted. In privacy-sensitive deployments, raw visual data may be discarded immediately after processing, ensuring that only non-identifiable, structured observations persist in the system. This approach directly addresses the data minimisation principle central to the General Data Protection Regulation and reduces long-term storage costs. Collectively, these modality-specific processing pipelines convert diverse sensor observations into a cohesive analytical data representation that facilitates cross-modality correlation and spatiotemporal analysis. The platform facilitates integrated querying and analysis across diverse sensing modalities by standardising measurement formats and linking all observations with geospatial and session-level metadata.

### 4.3. Governance Framework

The governance framework establishes the administrative rules and enforcement mechanisms that regulate data ownership, access rights, and operational responsibilities across the platform. The framework is implemented through a hierarchical role-based access control model that operates at three organisational levels: system, provider, and dataset.

At the system level, platform administrators manage global configuration, define provider accounts, allocate computational and storage resources, and establish system-wide policies governing data retention, processing standards, and security requirements. System administrators do not, by default, have access to provider-owned data, ensuring a clear separation between infrastructure management and data governance.

At the provider level, each data provider is represented as an autonomous organisational entity with dedicated administrative roles. Provider administrators manage their own fleet registrations, sensor configurations, and user accounts. They define access policies that specify which users or consumer groups may query, download, or analyse data originating from their vehicles. Provider-level policies can impose restrictions based on temporal scope, spatial extent, data modality, or processing level, enabling fine-grained control over data sharing without requiring platform-wide policy changes.

At the dataset level, data managers oversee the quality, completeness, and lifecycle of individual data collections. They are responsible for reviewing processing outputs, annotating datasets with contextual metadata, and authorising the release of data products to approved consumers. Data consumers, in turn, interact with the platform exclusively through the governed API layer and have access only to those datasets for which the provider and the dataset governance hierarchy have granted explicit authorisation.

The governance framework enforces all access decisions at the point of data retrieval. When a consumer submits a query, the access control engine evaluates the request against the applicable provider policies, dataset permissions, and the consumer’s assigned roles before returning results. Denied requests are logged with the denial reason, and all successful data accesses are recorded in immutable audit logs that capture the requesting identity, timestamp, query parameters, and the volume of data returned. These audit records support regulatory compliance, enable retrospective accountability assessments, and provide an evidentiary basis for resolving disputes over data use.

Access control enforcement is implemented at both the API and database layers. Authenticated requests include token claims containing the provider_id and role_id attributes. API-layer filtering constrains query scopes before execution, while database-level constraints (including provider-scoped indexing and query parameterisation) prevent cross-provider data access. This dual-layer enforcement mitigates risks associated with application-layer misconfiguration.

Privacy governance extends beyond access control to encompass the entire data lifecycle. Retention policies, defined at the provider and system levels, specify maximum storage durations for each data modality and processing level. Automated lifecycle management routines periodically evaluate stored data against applicable retention policies and execute scheduled deletions or archival operations. For visual data, retention policies distinguish between raw imagery and derived metadata, permitting indefinite retention of structured detection records while enforcing strict time-limited retention of original frames. This differentiated approach balances analytical utility with privacy obligations, ensuring that the platform remains compliant with evolving regulatory requirements without sacrificing the long-term value of accumulated environmental and urban observations.

### 4.4. Privacy Risk Mitigation Analysis

The primary threat model considered includes (i) unauthorised cross-provider data access, (ii) long-term retention of personally identifiable visual information, and (iii) interception or tampering during data transmission. Mitigation strategies include provider-scoped access control, retention-based deletion of raw imagery, encrypted transmission channels, and immutable audit logging.

The architectural separation of raw imagery from derived, structured metadata reduces the long-term storage of personally identifiable information. In deployments where raw frames are deleted immediately after object detection, only anonymised metadata (object class, bounding box coordinates, confidence scores, geolocation) persists. This approach aligns with data minimisation principles under GDPR and reduces storage requirements compared to indefinite video retention. For example, a 15-min 100 MB video segment yields structured detection metadata that occupies 1–5% of the original storage volume, depending on scene complexity. This architectural enforcement of deletion policies reduces regulatory exposure by ensuring that identifiable visual content does not persist beyond operational necessity, thereby minimising compliance risks associated with long-term storage.

Unlike post hoc anonymisation approaches, this design prevents long-term retention of raw identifiable visual content at the architectural level.

## 5. System Implementation and Deployment

This section describes the concrete implementation of the architecture presented in Section 3 and Section 4, detailing the technology choices, service composition, and deployment configuration adopted for the research prototype. The implementation follows a fully containerised, open-source approach and has been validated through a pilot deployment with multiple vehicles and data providers.

### 5.1. Deployment Infrastructure and Technology Stack

**Figure 2:**
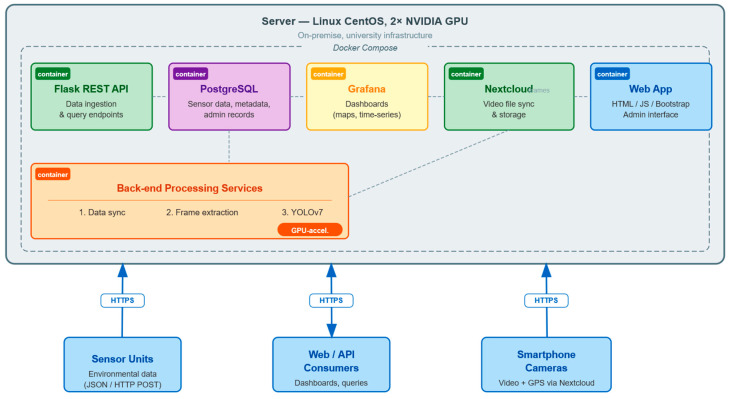
Containerised deployment topology of the research prototype. All platform services are packaged as Docker containers and orchestrated with Docker Compose on a single on-premises server running CentOS Linux and equipped with two NVIDIA GPUs. External clients access the platform through HTTPS-secured endpoints.

The platform is deployed on a single on-premises physical server at the university, running CentOS Linux 7, and equipped with two RTX 6000 GPUs (NVIDIA Corporation, Santa Clara, CA, USA) dedicated to computationally intensive visual data processing tasks. All platform services are packaged as Docker containers and orchestrated using Docker Compose (Figure 2), which defines the complete service topology, inter-container networking, and shared storage volumes in a single declarative configuration file. This approach enables reproducible deployments and simplifies service dependency management during the research phase. For production-scale deployments involving larger vehicle fleets and higher data throughput, migrating to a container orchestration platform such as Kubernetes is anticipated to provide automated horizontal scaling, self-healing, and rolling updates.

The core technology stack comprises open-source components summarised in Table 2. All external communication is secured through HTTPS with valid certificates.

The single-server deployment was selected to evaluate architectural viability under constrained infrastructure conditions. This configuration represents a lower-bound scenario for computational capacity, demonstrating that the proposed architecture does not require distributed clusters for moderate fleet sizes. The selected technology stack directly reflects the architectural principles defined in Section 3. Containerisation supports modularity and scalability; PostgreSQL with PostGIS enables geospatial indexing aligned with multimodal data; and GPU-accelerated inference supports the privacy-by-design visual abstraction pipeline.

The implementation of Docker Compose in the research prototype was mostly driven by the necessity for reproducible experimentation and streamlined deployment management during the pilot phase. The architectural design is not contingent upon a single-node deployment. All system services are executed as autonomous, containerised microservices with stateless processing components and externally controlled storage, making them natively compatible with container orchestration platforms such as Kubernetes or Docker Swarm. Thus, the prototype deployment signifies a basic infrastructure setup aimed at assessing architectural viability rather than the platform’s maximum scalability potential.

### 5.2. Data Ingestion Services

**Figure 3:**
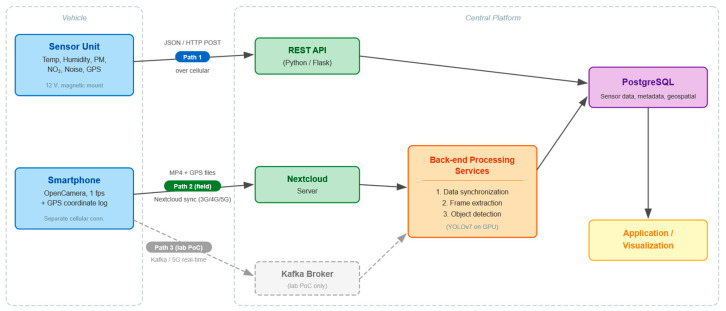
Data ingestion pathways from vehicle-mounted sensing units to the central platform. Structured sensor measurements are transmitted in real time via the REST API (Path 1). Visual data are synchronised through the Nextcloud-based store-and-forward pathway (Path 2, used in field deployment). An Apache Kafka pathway for near-real-time streaming (Path 3) was validated in laboratory conditions. All pathways converge at the backend processing services.

The platform implements two complementary data ingestion pathways (Figure 3), each addressing distinct operational requirements for the data modalities collected by vehicle-mounted sensors.

Structured sensor measurements—encompassing meteorological parameters, air quality metrics, and acoustic noise levels—are transmitted from the vehicle-mounted sensor unit to the platform through a REST API endpoint. The sensor unit is a single integrated hardware enclosure containing all environmental sensors, mounted on the vehicle exterior using magnetic fasteners and powered by the vehicle’s 12-volt battery. It communicates over an independent cellular connection, transmitting JSON-encoded data packages via HTTP POST requests. Each JSON message contains measurement values from all co-located sensors at a specific geographic position and point in time, along with geospatial coordinates, timestamps, and device-identification metadata. The Flask-based API validates incoming messages against the expected schema, performs integrity checks, and persists records in a PostgreSQL database.

Visual data acquisition uses a store-and-forward approach via a private Nextcloud instance. An Android smartphone running the open-source OpenCamera application captures image frames at 1 frame per second, along with a parallel log of GPS coordinates for each frame. Video segments are stored locally on the device as approximately 100-megabyte files, representing 15 to 25 min of recording. The mobile Nextcloud client application synchronises these files to the central Nextcloud server when sufficient cellular connectivity is available, ensuring reliable data transfer even over intermittent or low-bandwidth connections, such as 3G or 4G networks.

As a complementary pathway, Apache Kafka was evaluated in laboratory conditions for near-real-time streaming of video frames over 5G networks. The Kafka-based pipeline demonstrated successful real-time data delivery; however, the Nextcloud-based approach was selected for field deployment due to its greater resilience to connectivity interruptions, as locally buffered data are transmitted in compressed form once sufficient bandwidth becomes available. The architecture supports both pathways, allowing operators to select the ingestion method that best meets their connectivity and latency requirements.

**Figure 4:**
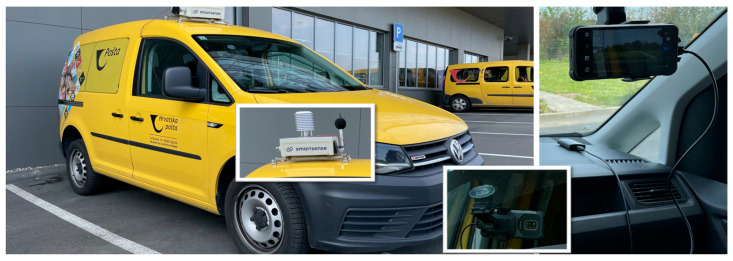
Vehicle-mounted data acquisition hardware. The integrated environmental sensor unit, comprising meteorological, air-quality, and acoustic sensors, is mounted to the vehicle exterior via magnetic mounts and powered by the vehicle’s 12 V battery, and an Android smartphone running the OpenCamera application is positioned inside the vehicle cabin to capture forward-facing images at 1 frame per second, along with a parallel GPS coordinate log. Each device communicates independently over separate cellular connections during the research phase.

During the research phase, the vehicle-mounted sensor unit and the smartphone camera operated on separate cellular connections (Figure 4), enabling independent testing and debugging of each data stream. For production deployments, consolidation to a single cellular gateway per vehicle is recommended to reduce hardware complexity and communication costs.

### 5.3. Backend Processing Services

**Figure 5:**
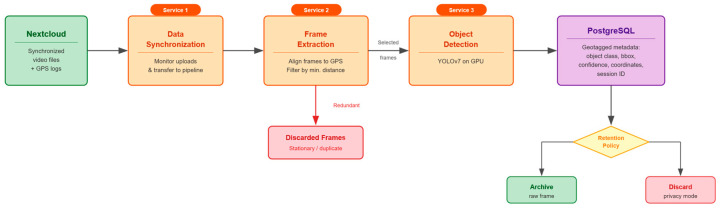
Visual data processing pipeline. Synchronised video files are parsed alongside their corresponding GPS coordinate logs. Frames are extracted and filtered using a configurable minimum-distance parameter to remove spatially redundant captures. Selected frames are submitted to the object detection model running on NVIDIA GPUs. Detected objects are geotagged and stored as structured metadata records in the PostgreSQL database. Retention policies determine whether original image frames are preserved or discarded after metadata extraction.

Three background processing services operate on the server (Figure 5), each managed through the web-based administrative interface, which allows authorised operators to start, stop, and monitor service status without command-line access.

The first service handles data synchronisation, monitoring the Nextcloud storage for newly uploaded video files and transferring them into the processing pipeline. The second service extracts video frames and parses synchronised video files alongside their corresponding GPS coordinate logs. It aligns each extracted frame with its geographic position. It applies a configurable minimum distance threshold to filter out redundant frames, such as those captured while the vehicle is stationary in traffic or at intersections. This parameterisation operates exclusively on the extracted frames used for analysis. It does not modify the original video recordings, enabling repeated re-extraction with different sampling configurations as analytical requirements evolve.

The third service performs object detection on the selected frames using the YOLOv7 model [40], which runs on the server’s NVIDIA GPUs. In the context of this research, YOLOv7 serves as a representative off-the-shelf inference component to demonstrate the feasibility of GPU-accelerated, real-time visual abstraction within the ingestion pipeline —not as a domain-optimised detector. While the initial prototype did not include a formal accuracy evaluation, a post-hoc precision-focused assessment was conducted. The architecture was intentionally model-agnostic: production deployments may substitute fine-tuned or purpose-trained models targeting specific object categories without requiring modifications to the surrounding system architecture. Optimising detection accuracy for particular urban sensing scenarios is identified as a direction for future domain-specific work. Detected objects are geotagged using the frame’s associated coordinates and persisted as structured metadata records in the PostgreSQL database, linked to the originating sensing session, vehicle, and provider.

### 5.4. Front-End Application and Visualisation

The web application serves as the central administrative and monitoring interface for the platform. It exposes functionality organised into five operational domains: system management, oversight of data acquisition and dissemination, client account management, sensor and device configuration, and vehicle provider administration. Through this interface, authorised users manage provider registrations, configure sensor parameters, control background processing services, and review system status.

Data visualisation is delivered through Grafana dashboards embedded within the web application. Two principal data categories are presented: discrete observations, comprising geotagged detected objects displayed on interactive map layers, and continuous measurements, including time-series plots of air quality parameters, meteorological readings, and acoustic noise levels. Both categories are accessible interactively via the web interface and programmatically via the REST API, enabling integration with external analytic tools and third-party applications.

Access control follows the role-based model described in Section 4.3 and is implemented via token-based authentication over encrypted connections. The platform supports flexible authentication mechanisms—including JSON Web Tokens, OAuth 2.0, and session-based approaches—allowing system operators to select the method best suited to their security requirements or existing identity management infrastructure. Table 3 summarises the permissions enforced for each principal role in the platform’s access control layer.

The role-based access control approach outlined in Table 4 delineates distinct duties among system administrators, provider operators, data managers, and data consumers. By consistently enforcing these rights across both the application and data layers, the platform enables safe multi-provider collaboration while maintaining data ownership limits and governance regulations.

### 5.5. Pilot Deployment and Validation

The platform was validated through a phased pilot deployment. During the preparation and experimentation phase, two private vehicles were equipped with sensor units and smartphones to establish baseline system functionality and refine the data acquisition workflow. Subsequently, two vehicles operated by a commercial delivery provider collected data continuously for two months, testing system reliability under realistic operational conditions with independent fleet management. Table 4 summarises the configuration and outcomes of each deployment phase.

Nextcloud-based ingestion was validated using concurrent data streams from real vehicles, whereas the Kafka-based pathway was tested with two simultaneous streams under laboratory conditions. The system demonstrated stable operation throughout the extended pilot period, confirming the viability of the containerised architecture for sustained multi-vehicle, multi-provider data collection and showing that governed multimodal vehicle sensing can be implemented without reliance on large-scale public cloud infrastructure. The pilot deployment was deliberately executed at a moderate fleet scale to assess the architecture’s operational viability under realistic yet controlled conditions. The main aim of this phase was to validate the reliability of multimodal data intake, the robustness of the processing pipelines, and the accuracy of the governance mechanisms for managing data from multiple independent sources. Although the number of cars operating simultaneously was limited, the implementation nevertheless encompassed data streams from many organisational domains, facilitating verification of provider-level data isolation and access control systems.

### 5.6. Governance Validation Tests

A set of validation tests was conducted to assess the efficacy of the multi-provider governance mechanisms installed on the platform, focusing on data isolation and access control enforcement.

Initially, cross-provider access attempts were emulated by generating API requests using authentication tokens associated with multiple provider identities. The tests confirmed that the API layer appropriately limited data retrieval based on provider identifiers embedded in the authentication context. Secondly, database-level row-level security (RLS) policies were verified by running direct queries under constrained roles. The database setup prevented the retrieval of records linked to other providers, thereby maintaining tenant isolation even in the event of misconfigured application-layer access controls. Third, metadata access patterns were examined to assess the danger of indirect cross-provider inference via aggregated enquiries. The query results verified that the system revealed only data subsets permitted by providers and that the foundational access controls obstructed metadata linkage across organisational domains.

The validation tests verify that the governance model established in the design ensures effective enforcement of provider-level data isolation at both the application and database layers.

### 5.7. Privacy and GDPR Considerations

The system architecture was developed in accordance with privacy-by-design principles to mitigate potential risks associated with the processing of visual and environmental data collected in urban settings. A qualitative privacy risk evaluation was conducted, focusing on three main risk categories: identifiable visual data, cross-provider data exposure, and prolonged retention of raw sensor data.

The architecture mitigates these dangers through various structural techniques. Initially, raw video streams are not retained for extended periods; instead, visual observations are converted into structured information records during the intake phase. This design markedly diminishes the likelihood of retaining identifiable visual data. Secondly, provider-level data isolation technologies guarantee that datasets from distinct organisational entities are logically segregated, hence preventing unauthorised cross-tenant access. Third, access to stored data is governed by role-based access control policies that limit retrieval operations to permitted roles and provider domains.

The platform does not handle personal identity data; however, these architectural measures further mitigate possible privacy threats and uphold the data minimisation and access control standards established by the General Data Protection Regulation (GDPR).

### 5.8. Performance Evaluation

A performance evaluation is undertaken during the pilot deployment to examine the operational benefits of the proposed design. The assessment emphasises several system-level measures pertinent to mobile sensing infrastructures: visual inference latency, ingestion reliability, database query speed, API response times, and storage efficiency achieved through ingestion-time visual abstraction. These parameters jointly define the computing efficiency, scaling potential, and operational robustness of the proposed platform.

#### 5.8.1. Frame Processing Performance

Visual data processing was evaluated on the on-premise server equipped with two NVIDIA GPUs. Using the pretrained YOLOv7 model, the average object detection inference time per extracted frame was approximately 200 ms under nominal load conditions. Peak processing time during concurrent workload spikes reached 240 ms, while the minimum observed inference time was 170 ms.

With a sustained frame arrival rate of 1 frame per second per vehicle, the system maintained real-time processing capability for multiple concurrent vehicle streams without backlog accumulation. GPU utilisation during continuous processing averaged 68%, with short bursts reaching 82% under parallel extraction and inference operations.

#### 5.8.2. Object Detection

To assess the analytical validity of the visual processing pipeline, the pretrained YOLOv7 model (COCO dataset) was evaluated on a sampled subset of frames collected during the pilot deployment. No task-specific fine-tuning was performed in the current prototype implementation.

Object detection was performed with an input resolution of 640 × 360 pixels, a confidence threshold of 0.5, and a non-maximum suppression threshold of 0.45. Detections below the defined confidence threshold were excluded from metadata storage.

Although the model was not fine-tuned on locally collected data, the observed detection performance is sufficient for applications such as aggregate counting, infrastructure monitoring, and traffic density estimation. The architecture permits seamless substitution or retraining of detection models without structural modification, enabling further accuracy improvements in future deployments. To assess whether the generated object-level metadata is sufficiently reliable for downstream analytical use, a targeted quantitative evaluation was conducted on a representative subset of detections.

#### 5.8.3. Precision-Based Evaluation of Detection Metadata

A precision-focused evaluation was conducted on a stratified random sample of detection records from the pilot deployment dataset to assess the reliability of the object-level metadata generated by the visual processing pipeline. The assessment focused on precision—the ratio of stored detections that accurately match identified objects—as the metric most pertinent to metadata reliability, since a false positive generates an incorrect entry in the database. In contrast, a missed detection (affecting recall) merely results in a missing record that does not undermine the integrity of subsequent analyses.

The trial deployment amassed 88,041 object-detection records across six item categories using the standard pretrained YOLOv7 model. A stratified random sample of 250 detection records was extracted from the database: 50 records for each of the four predominant classes (vehicle, truck, person, and traffic light), 30 for stop sign, and 20 for bicycle, mirroring the relative frequency of each class in the dataset. Each sampled record underwent visual inspection by a human assessor, who evaluated the matching image frame as either a true positive (accurate detection) or a false positive (inaccurate detection). Table 5 encapsulates the findings.

The findings indicate significant discrepancies in precision among item categories. The car class attains an exceptional precision of 0.98, attributable to the robust representation of passenger vehicles in the COCO training set and their visually distinguishing characteristics in dashcam footage. Truck and individual detections achieve precision scores of 0.80 and 0.82, respectively. The precision for traffic lights and bicycles is 0.66 and 0.65, respectively; the stop sign class demonstrates significantly low precision at 0.20.

The qualitative study of false-positive records identifies three primary categories of inaccuracy. The first and most significant issue is the domain mismatch between the COCO training distribution and the European metropolitan setting experienced after deployment. The COCO “stop sign” class is overwhelmingly represented by the octagonal red signs used in North America. Within the European road environment of the pilot deployment, the model consistently misidentified round regulatory signs—such as speed limit signs, one-way signs, traffic prohibition signs, and the reverse sides of circular signs—as stop signs, resulting in 24 of 30 sampled stop-sign records being false positives. Likewise, three of the seven bicycle false positives pertained to motorcycles, a classification boundary that aligns with COCO category boundaries rather than indicating a fundamental detection error.

The second category of errors pertains to camera artifacts. Rearview mirrors, partially within the camera’s field of view, were erroneously identified as traffic lights on six occasions, constituting over one-third of the false positives for traffic lights. Contaminated or rain-marked windshields, solar glare, and excessive exposure resulted in erroneous detections across various categories. These artifacts result from the uncontrolled mounting conditions characteristic of vehicle-based data-gathering situations, in which cameras are operated by fleet vehicle drivers lacking specialized expertise.

The third category includes environmental visual ambiguities, such as shadows erroneously identified as individuals (three occurrences), reflections, and visually analogous urban elements like billboards, hedges, and building facades.

Excluding the stop sign category, which indicates a distinct training-domain discrepancy rather than a constraint in detecting capabilities, precision for the other five categories increases to 0.80 (176 true positives from 220 evaluated records). This outcome demonstrates that the pretrained general-purpose model generates metadata with adequate reliability for aggregate spatial analyses, including traffic density estimation and pedestrian activity mapping, where the quantity of accurate detections mitigates individual false positives. For applications that require enhanced per-record accuracy or the identification of region-specific object categories (e.g., European road signs), the modular design of the processing pipeline enables replacement of fine-tuned or specialized models without altering the surrounding infrastructure.

Recall was not independently assessed in this work, since it would necessitate comprehensive annotation of all items within a collection of raw frames—a significantly more resource-demanding endeavour. The evaluation scope was intentionally confined to precision, directly addressing the trustworthiness of the metadata entries maintained in the database for downstream use. The pretrained YOLOv7 model provides per-detection confidence scores; however, these values were not preserved in the study prototype’s database structure.

Persisting per-detection confidence scores and training region-specific object detection models (e.g., for European road signage) are identified as priorities for improving metadata precision in production deployments.

#### 5.8.4. Concurrent Stream Validation

Concurrent ingestion performance was evaluated in laboratory conditions using Apache Kafka-based near-real-time streaming. The system successfully handled 2 simultaneous high-definition video streams over a 5G connection without packet loss or processing failure. During these tests, the average end-to-end frame delivery latency (from the vehicle to the processed metadata storage) was measured at 420 ms.

Although the field deployment relied on the Nextcloud-based store-and-forward mechanism, laboratory validation confirmed that the architecture can support near-real-time ingestion under high-bandwidth conditions.

#### 5.8.5. Pilot Duration and Stability

The field pilot deployment operated continuously for approximately 2 months, involving two commercial delivery vehicles. During this period:

- Total structured sensor records ingested: 5.4 million records;
- Total video segments synchronised: 1319 files;
- Total extracted frames processed: 1.4 million frames;
- System uptime: 99.2%;
- Recorded data loss incidents: 0.

The results demonstrate that the platform effectively maintained continuous multimodal data collecting and processing under actual operational conditions during the trial period. The elevated system uptime, the lack of documented data-loss incidents, and the effective handling of substantial volumes of sensor data and video frames illustrate the architectural stability and the dependability of the ingestion and processing pipelines. The trial deployment, despite its limited number of cars, validates the practicality of the proposed platform for extended mobile sensing campaigns.

#### 5.8.6. Data Ingestion Reliability

Sensor data transmitted via REST API endpoints was validated against predefined JSON schemas. Throughout the full pilot, the schema validation failure rate remained below 0.4%, primarily due to malformed test submissions during early experimentation. No valid production data packets were discarded after system stabilisation.

The store-and-forward synchronisation mechanism demonstrated reliable recovery from intermittent cellular connectivity disruptions, with an average synchronisation delay of 6.5 min after reconnection.

#### 5.8.7. API Response Latency

API performance was evaluated through repeated query tests on both time-series sensor data and object-detection metadata. Under nominal load conditions (single-user dashboard queries), the average API response time for typical filtered queries (spatial + temporal constraints) was 120 ms.

Under simulated concurrent access (10 parallel query requests), average response time increased to 310 ms, remaining within acceptable interactive dashboard thresholds. The maximum observed latency during stress testing reached 480 ms.

#### 5.8.8. Database Query Performance

PostgreSQL query performance was evaluated for representative workloads:

- Retrieval of 24-h air quality time-series (single vehicle): 125 ms;
- Retrieval of geotagged object detections within a 5 km radius and 7-day interval: 250 ms;
- Aggregated count queries over 1 million records: 280 ms;
- Database CPU utilisation during peak querying remained below 55%, indicating available capacity for additional scaling.

The results indicate that the database layer can effectively handle standard analytical workloads with minimal query delay, even when processing datasets comprising millions of records. The recorded response times enable near-real-time data analysis via dashboards and API-driven analytical queries. At the same time, the moderate CPU utilisation suggests that additional workloads could be supported without significant infrastructure enhancements. The assessment reveals that the database design delivers adequate performance for interactive urban sensing analysis and operational monitoring.

#### 5.8.9. Storage Efficiency

In privacy-preserving deployments in which raw image frames were deleted after metadata extraction, storage reduction was substantial. A typical 100 MB video segment produced structured metadata that occupied approximately 2–5 MB, resulting in a storage reduction of roughly 95% compared to retaining the raw data indefinitely.

While the evaluation was conducted at a moderate fleet scale, the observed resource utilisation indicates the capacity for horizontal scaling via container replication and orchestration-based load balancing in larger deployments.

To provide a consolidated overview of the quantitative evaluation results, Table 6 summarises the principal performance metrics observed during laboratory validation and the two-month pilot deployment. These metrics collectively characterise the computational efficiency, ingestion reliability, query responsiveness, and storage optimisation achieved by the proposed architecture under realistic operating conditions.

The results illustrate multiple practical advantages of the suggested architecture. The GPU-accelerated visual processing pipeline achieves real-time inference performance suitable for ongoing vehicle-based sensing applications. The asynchronous ingestion architecture enables reliable data collection despite sporadic network access, as evidenced by the lack of irreversible data loss during the pilot deployment. The segregation of unprocessed visual data from structured metadata significantly enhances storage efficiency, reducing long-term storage needs by roughly 95% compared to the perpetual preservation of raw video. The measured latencies of API and database queries demonstrate that the platform can support interactive analytical applications and real-time monitoring dashboards without requiring dispersed cloud infrastructure at moderate fleet scales.

## 6. Application Scenarios

This section presents illustrative application scenarios that demonstrate how the platform architecture, data acquisition mechanisms, and processing pipelines described in the preceding sections can be applied to real-world urban monitoring and smart city use cases. Each scenario leverages continuous environmental sensor measurements and geotagged visual observations collected by vehicle-mounted sensing units, processed by backend services, and delivered to stakeholders via the governed access and visualisation layers.

### 6.1. Environmental Quality Monitoring

The most direct application of the platform is continuous environmental monitoring along urban vehicle routes. As equipped vehicles traverse their regular operational paths, the integrated sensor unit captures air quality measurements—including particulate matter, nitrogen dioxide, ozone, and carbon monoxide—alongside meteorological parameters such as temperature, humidity, atmospheric pressure, and wind speed. Simultaneously, the acoustic sensor records sound pressure levels at each geographic point. During the pilot deployment, air quality measurements collected along repeated delivery routes demonstrated consistent spatial variation near high-traffic corridors, illustrating the practical feasibility of route-based environmental profiling.

Because these measurements are transmitted as geotagged, timestamped records through the REST API, the platform accumulates fine-grained spatial and temporal environmental profiles. Over successive traversals of the same routes, the system builds longitudinal datasets that reveal how pollution levels, microclimatic conditions, and noise exposure vary by location, time of day, season, and proximity to emission sources, such as construction sites, industrial facilities, or high-traffic corridors. Municipal authorities, environmental agencies, and public health researchers can access these datasets via Grafana-based visualisation dashboards or programmatically via the API, enabling evidence-based decision-making for urban planning, noise regulation enforcement, and pollution mitigation strategies. The multi-provider governance model ensures that data collected by different fleet operators remains attributable and independently manageable while still supporting aggregated cross-provider analyses where authorised.

### 6.2. Traffic and Road Infrastructure Assessment

The visual data processing pipeline enables a distinct class of applications for assessing road and traffic infrastructure. With appropriately trained object detection models, the system can identify and classify not only moving objects such as vehicles, pedestrians, and cyclists, but also static infrastructure elements captured in geotagged image frames. This capability enables the systematic detection of damaged or obscured traffic signs, malfunctioning traffic lights, degraded road markings, and road-surface defects such as potholes or significant cracks. Because each detection is automatically geotagged and stored as structured metadata in the database, municipal road maintenance departments can generate spatial inventories of infrastructure conditions across the entire area covered by participating vehicle fleets. Repeated route coverage enables temporal comparison, allowing operators to track the progression of pavement deterioration or verify that reported defects have been addressed. Traffic flow analysis is another application in this domain: by counting and classifying vehicles by type along specific road segments across multiple passes, the platform produces traffic density estimates that complement or replace fixed counting stations. These estimates can inform decisions on road capacity planning, weight-restriction enforcement, and the allocation of maintenance resources to the most heavily utilised segments. The role-based access control model ensures that raw visual data and derived traffic analytics are shared only with authorised stakeholders, in accordance with the provider’s defined policies. In such applications, provider-level governance ensures that raw imagery remains under fleet operator control, while municipal authorities may receive only aggregated or anonymised detection statistics.

### 6.3. Smart City Maintenance and Urban Governance

Beyond traffic infrastructure, the platform supports broader urban maintenance and governance applications that benefit from regular, geographically distributed visual surveys. Urban greenery management is a compelling example: by training models to detect and classify trees and vegetation, the system can produce geotagged inventories of street trees, estimate canopy coverage, and flag visual indicators of poor plant health, such as extensive leaf loss or discolouration. Such inventories support municipal arboriculture programs and urban heat island mitigation planning. Parking space occupancy can be estimated from visual observations along routes, capturing spatial and temporal patterns of parking demand without dedicated sensors at individual spaces. Similarly, the concentration and flow patterns of specific road user categories—bicycles, electric scooters, and motorcycles—can be quantified through repeated visual observations, informing the planning of cycling infrastructure, micro-mobility regulations, and pedestrian safety interventions. In all these scenarios, the asynchronous multi-provider architecture offers a practical advantage: municipalities need not operate dedicated monitoring fleets; instead, they can integrate data from commercial delivery providers, public transit operators, or municipal service vehicles that already traverse urban areas in the course of routine operations. The governance framework ensures that each participating provider retains control over their data while the municipality, as a data consumer, accesses aggregated and anonymised results through the governed API layer.

### 6.4. Electromagnetic Environment Mapping

The modular design of the vehicle-mounted sensor unit enables integration of additional measurement capabilities beyond those of the environmental sensors in the current research prototype. One such extension is the characterisation of the urban electromagnetic environment. By incorporating radio-frequency measurement modules or mobile-network diagnostic instruments into the sensor enclosure, participating vehicles can record electromagnetic field strengths and mobile-network performance indicators, such as signal strength, connection type, latency, and throughput, along their operational routes. These measurements, transmitted through the same REST API ingestion pathway and stored alongside other sensor data in the PostgreSQL database, enable the construction of detailed spatial maps of wireless coverage quality and electromagnetic exposure levels. Such data could help telecommunications operators identify coverage gaps, optimise base station placement, and validate network performance under real-world conditions. Public health authorities and urban planners may use electromagnetic exposure maps to assess compliance with exposure guidelines and inform the siting of sensitive facilities such as schools and hospitals. Because the ingestion and storage pipelines treat all structured sensor data uniformly, each measurement is represented as a geotagged, timestamped record associated with a provider, device, and session. Adding new sensor modalities requires no architectural changes; only registering new data fields in the schema and configuring the corresponding visualisation dashboards are required.

The scenarios presented in this section illustrate the breadth of applications enabled by the combination of multimodal data acquisition, automated visual analysis, and governed multi-provider data management. The modular architecture ensures that new sensing modalities and analytical objectives can be accommodated without structural modifications, positioning the platform as a versatile foundation for diverse smart city monitoring and urban governance initiatives.

Collectively, these scenarios demonstrate that the architectural contribution is not tied to a single analytical task but instead provides a governance-aware sensing substrate. The key innovation is the ability to enable cross-organisational participation without compromising provider autonomy. This structural flexibility differentiates the platform from application-specific sensing deployments.

## 7. Limitations

While the proposed architecture demonstrates practical feasibility and operational stability, several limitations must be acknowledged. The limitations discussed below are categorised into architectural scalability constraints, scope of empirical validation, depth of governance validation, and model-level analytical constraints. These limitations inform the interpretation of the results and guide prioritised future research directions.

- First, the pilot deployment was conducted on a single on-premise server within a university-controlled environment. Although containerised microservices and GPU acceleration were successfully validated, the evaluation did not include distributed multi-node orchestration or large-scale horizontal scaling under high vehicle density. Consequently, system behaviour under substantially larger fleets—such as tens or hundreds of concurrent vehicles—was not empirically tested. While resource utilisation trends suggest linear scalability, non-linear contention effects in distributed orchestration environments cannot be excluded without large-scale empirical validation. The present assessment thus represents a proof-of-concept infrastructure setup rather than a comprehensive distributed private cloud implementation.
- Second, the number of concurrently active vehicle streams during validation remained moderate. The field deployment involved four vehicles, and laboratory stress testing of real-time video ingestion was limited to two simultaneous Kafka streams. As a result, extensive multi-provider contention scenarios involving numerous concurrently operating vehicles were not assessed in this study and represent a significant avenue for future validation.
- Third, performance evaluation focused primarily on system-level operational metrics, including processing latency, ingestion reliability, and query responsiveness. The study did not conduct a comparative benchmark against alternative architectures, such as public cloud deployments or fully edge-based processing systems. Therefore, relative efficiency gains compared to other architectural paradigms remain to be empirically quantified.
- Fourth, the object detection component relied on a pretrained YOLOv7 model without task-specific fine-tuning on locally collected datasets. Although this approach demonstrates architectural generality and plug-in model flexibility, detection accuracy under diverse environmental conditions (e.g., varying illumination, weather, or camera positioning) was not systematically evaluated. Future research should incorporate formal accuracy assessment and model adaptation strategies.
- Fifth, privacy-by-design principles were operationalised through architectural separation of raw imagery and structured metadata, along with configurable retention policies. However, the study did not include a formal privacy risk assessment or adversarial threat modelling analysis. While data minimisation is enforced at the structural level, additional techniques such as differential privacy, federated learning, or cryptographic access controls were not implemented in the current prototype. Additionally, no independent security penetration testing was conducted during the pilot phase.
- Sixth, interoperability testing across heterogeneous sensor vendors was limited. Although the ingestion layer is schema-based and modality-agnostic, the pilot deployment used a single integrated environmental sensor enclosure and a standardised camera configuration. Broader validation across multiple hardware platforms would further demonstrate architectural generality.
- Finally, the evaluation period, which spans approximately two months, does not capture seasonal environmental variation or long-term operational drift in sensor calibration. Extended longitudinal deployments would be required to assess durability, maintenance overhead, and calibration stability over annual cycles.

The current validation, therefore, reflects moderate-scale deployment conditions (≤4 vehicles, ≤3 concurrent real-time streams) rather than metropolitan-scale fleet density.

Despite these limitations, the results demonstrate that the proposed governance-aware private cloud architecture is technically viable for multi-provider vehicle-mounted multimodal sensing and provides a structured foundation for future large-scale expansion. Furthermore, these limitations define the operational boundary of the present validation and motivate structured future investigation.

## 8. Future Works

To address the scalability and orchestration constraints identified in Section 7, future work will prioritise validating distributed orchestration and adaptive edge–cloud workload distribution. The current approach centralises visual processing in a private cloud environment, while future iterations will explore dynamic workload distribution among on-vehicle devices, roadside infrastructure, and cloud resources. That entails assessing task offloading solutions for frame preprocessing, object detection, and metadata aggregation to optimise bandwidth utilisation, minimise end-to-end delay, and enhance resilience in high-vehicle-density scenarios.

A second research avenue concerns integrating sophisticated privacy-preserving measures into the processing pipeline. Future work will investigate integrating federated learning into object identification training to enable cross-provider model enhancement without centralised data transfer. Moreover, differential privacy mechanisms will be used for aggregated metadata queries to reduce re-identification risk in multi-provider analytics while maintaining statistical utility.

Semantic interoperability and data standardisation are another essential domain. Implementing unified ontologies and standardised data models for sensors, vehicles, detected entities, and environmental indicators will enhance cross-platform interoperability and enable inter-city or cross-domain applications. Aligning schemas with current smart city standards will promote extensibility outside individual institutional contexts.

Comprehensive empirical validation is also necessary. Large-scale deployments will evaluate horizontal scalability under urban fleet densities, assess the robustness of governance enforcement in high-density multi-provider contexts, and measure system stability under prolonged high-throughput operation. This research will provide quantitative scalability modelling and identify potential bottlenecks in distributed orchestration systems.

Subsequently, forthcoming research will concentrate on creating sophisticated data-driven applications and decision-support services based on the structured multimodal datasets generated by the platform. Instead of modifying the architectural core for specific applications, these advancements will leverage existing metadata abstractions and time-series data to support environmental trend modelling, infrastructure state assessment, traffic flow forecasting, and urban planning optimisation.

These research directions collectively aim to validate metropolitan-scale scalability, formalise privacy assurance mechanisms, and improve semantic interoperability across diverse sensing ecosystems.

## 9. Conclusions

This article presented and empirically validated a governance-aware private cloud architecture that enables scalable, multi-provider, vehicle-based multimodal sensing. The proposed architecture addresses four critical challenges in mobile IoT deployments: asynchronous data ingestion, integration of heterogeneous sensing modalities, privacy-sensitive visual processing, and enforceable multi-tier governance among independent stakeholders.

The findings demonstrate that RQ1 is addressed by an asynchronous, containerised ingestion and processing architecture that supports independent data providers under heterogeneous network conditions. RQ2 is addressed through structural provider isolation and dual-layer policy enforcement embedded within both the API and database layers, ensuring that governance is implemented as a core architectural property rather than delegated to application-level logic. RQ3 is addressed through ingestion-time visual abstraction and lifecycle-based retention policies that operationalise data minimisation directly within the system architecture.

The principal contribution of this work lies in embedding administrative governance and provider-level isolation directly within the system’s architectural layers. Role-based access control, provider-aware data segregation, and lifecycle-based retention policies collectively enforce data ownership and usage rights throughout the entire data lifecycle, from ingestion through retrieval. The privacy-by-design visual processing pipeline separates raw imagery from structured metadata early in processing, enabling substantial reductions in the long-term storage of potentially sensitive visual content while preserving analytical utility.

The proposed architecture was implemented using a fully open-source, containerised technology stack and validated through a two-month pilot deployment involving multiple vehicles and independent providers. Performance evaluation demonstrated real-time GPU-accelerated object detection, reliable asynchronous ingestion without data loss, stable operation across multiple providers, and low-latency API and database query performance suitable for interactive applications. Object detection validation confirmed that the visual processing pipeline produces analytically meaningful metadata supporting infrastructure monitoring, traffic estimation, and broader urban analytics tasks. The presented evaluation confirms that ingestion-time visual abstraction produces sufficiently reliable metadata for downstream analytical applications.

While large-scale distributed orchestration and comprehensive stress testing remain areas for further investigation, the results indicate that the architecture provides a resilient and adaptable foundation for privacy-aware vehicle-mounted sensing in smart city contexts. By unifying governance enforcement, privacy-by-design processing, and multimodal mobile sensing within a single architectural framework, this work establishes a replicable architectural paradigm for governed, privacy-aware, multi-actor urban sensing infrastructures suitable for metropolitan-scale deployment.

## Figures and Tables

**Table 1:** Comparison of the proposed platform with representative related systems across key architectural and functional dimensions. A checkmark (Y) indicates that the feature is explicitly addressed; a dash (-) indicates that it is absent or not discussed; a bullet (o) indicates partial or limited support.

Feature	Devarakonda et al. [2]	Apte et al. [3]	Ji et al. [5] (Survey)	Hull et al. [6]	Zanella et al. [21]	Catlett et al. [36]	Sanchez et al. [37]	Aberer et al. [38]	Eisenman et al. [39]	Ji et al. [11]	Zhong et al. [12]	Sruk et al. [13]	Marchesani et al. [34]	This Work
Vehicle-mounted mobile sensing	Y	Y	Y	Y	-	-	o	Y	o	Y	Y	Y	o	Y
Multimodal data (sensor + visual)	o	o	o	o	Y	Y	Y	o	o	Y	Y	Y	o	Y
Asynchronous store-and-forward ingestion	-	-	-	Y	o	o	-	o	Y	o	o	o	Y	Y
Multi-provider governance	-	-	o	-	o	o	o	o	-	-	-	-	Y	Y
Role-based access control (RBAC)	-	-	-	-	o	-	o	-	o	-	-	-	Y	Y
Privacy-by-design for visual data	-	-	-	-	-	Y	-	-	o	-	o	Y	Y	Y
Open-source technology stack	-	-	-	o	o	Y	o	Y	-	-	Y	o	o	Y
Containerised microservice deployment	-	-	-	-	-	o	-	-	-	-	Y	o	Y	Y
Pilot deployment with real vehicles	Y	Y	-	Y	-	-	o	Y	o	Y	o	o	Y	Y

**Table 2:** Open-source technology stack of the research prototype.

Component	Technology	Role
Database	PostgreSQL 14.5	Structured sensor data, detection metadata, geospatial records, and administration
REST API	Python 11.2/Flask 2.1.3	Data ingestion endpoints, query interfaces, and authentication
Front-end	HTML/JavaScript/Bootstrap	Administrative and monitoring web application
Visualisation	Grafana 9.3.1	Interactive dashboards (maps, time-series) embedded via iframes
Video file sync	Nextcloud 26.0.1	Store-and-forward video file synchronisation from vehicles
Real-time streaming (PoC)	Apache Kafka 7.0.1	Near-real-time video frame ingestion over 5G (lab validation)
Object detection	YOLOv7	Pretrained model for entity detection on extracted video frames
Video capture	OpenCamera 1.52 (Android)	1 fps image capture with parallel GPS coordinate logging
Containerisation	Docker 26.1.4/Docker Compose 2.33.1	Service packaging, orchestration, reproducible deployment
Server OS	CentOS Linux 7	Host operating system for the on-premise server
GPU acceleration	2x RTX 6000 GPU	Dedicated hardware for YOLOv7 inference
Transport security	HTTPS (TLS)	Encrypted communication for all external endpoints

**Table 3:** Role-based access control matrix.

Permission	System Admin	Provider Admin	Data Manager	Data Consumer
Global system configuration	Y	-	-	-
Provider account creation	Y	-	-	-
Resource allocation (compute, storage)	Y	-	-	-
System-wide retention/security policies	Y	-	-	-
Fleet and vehicle registration	-	Y	-	-
Sensor/device configuration	-	Y	-	-
User account management (own provider)	-	Y	-	-
Data access policy definition	-	Y	-	-
Data submission (ingest data)	-	Y	-	-
Processing service control (start/stop)	Y	Y	-	-
Dataset quality review and annotation	-	-	Y	-
Dataset release authorisation	-	-	Y	-
Query data via API/dashboards	-	-	Y	Y
Export authorised datasets	-	-	Y	Y
Access to provider-owned raw data	-	Y *	-	-
Audit log access	Y	Y +	-	-

* Provider administrators access only their own provider’s data. + Provider administrators can view audit logs only for their own provider scope. Y Feature is fully supported.

**Table 4:** Summary of the pilot deployment phases, including vehicle configurations, ingestion pathways, and validation outcomes.

Parameter	Phase 1: Preparation	Phase 2: Field Test
Purpose	Baseline functionality, workflow refinement	Reliability under operational conditions
Vehicles	2 private vehicles	2 commercial delivery vehicles
Provider type	Research team	Independent commercial fleet operator
Duration	Variable (iterative testing)	2 months continuous
Sensor unit	Environmental sensor enclosure (exterior mount)	
Camera	Android smartphone (OpenCamera, 1 fps)	
Cellular connections	Separate per device (sensor + camera)	
Ingestion: sensor data	REST API (JSON/HTTP POST)	
Ingestion: visual data	Nextcloud (store-and-forward)	
Kafka pathway	2 concurrent streams (lab only)	Not used in the field
System stability	Iterative debugging	Stable throughout 2 months

**Table 5:** Precision of the pretrained YOLOv7 object detection model evaluated on a stratified random sample of 250 detection records from the pilot deployment dataset. N (total) denotes the total number of detections of that class in the database; N (sample) is the number of records inspected.

Object Class	N (total)	N (Sample)	TP	FP	Precision
Car	38,328	50	49	1	0.98
Truck	17,899	50	40	10	0.80
Person	12,985	50	41	9	0.82
Traffic light	12,253	50	33	17	0.66
Stop sign	4629	30	6	24	0.20
Bicycle	1947	20	13	7	0.65
All classes	88,041	250	182	68	0.73

**Table 6:** Summary of key performance metrics.

Metric	Observed Value
Average object detection inference time	200 ms
Peak object detection inference time	240 ms
Concurrent Kafka video streams (lab validation)	2 streams
End-to-end frame delivery latency (Kafka, 5G)	420 ms
Total structured sensor records ingested (pilot)	5.4 million
Total extracted frames processed (pilot)	1.4 million
System uptime during pilot	99.2%
Recorded permanent data loss incidents	0
REST API average response time (single user)	120 ms
REST API response time (10 concurrent queries)	310 ms
PostgreSQL 24-h time-series query	125 ms
Geospatial object detection query (5 km, 7 days)	250 ms
Aggregated query over 1 million records	280 ms
Maximum database CPU utilisation (query peak)	55%
Metadata storage size per 100 MB video segment	2–5 MB
Approximate storage reduction (metadata vs. raw)	95%

## Data Availability

Restrictions apply to the datasets per the terms of the project funding.
